# Young Consumers’ Preferences for Nonalcoholic Beers: The Impact of Sensory Perception and Label Information

**DOI:** 10.1155/ijfo/9015139

**Published:** 2026-05-15

**Authors:** Dorota Klensporf-Pawlik, Inga Klimczak, Iga Rybicka, Anna Gliszczyńska-Świgło

**Affiliations:** ^1^ Department of Food Quality and Safety, Institute of Quality Science, Poznań University of Economics and Business, Poznan, Poland, put.poznan.pl; ^2^ Department of Technology and Instrumental Analysis, Institute of Quality Science, Poznań University of Economics and Business, Poznan, Poland, put.poznan.pl

**Keywords:** CATA, consumer perception, label, nonalcoholic beer, purchase intention, sensory quality

## Abstract

The growth of the nonalcoholic beer (NAB) market and diverse consumer preferences across nationalities prompted an evaluation of Polish young consumers’ perceptions of NAB (lager, India pale ale (IPA), wheat) and the impact of brand and label on product acceptance. The check‐all‐that‐apply (CATA) method and the correspondence analysis (CA) revealed that consumers perceived lager NABs over IPA and wheat beers, preferring refreshing, aromatic, and clear beers. Assessment of the impact of brand, attractiveness, and label clarity on NAB sensory perception in blind and informed conditions showed that some beers improved with brand information. The multiple linear regression (MLR) analysis showed that label attractiveness and clarity are good predictors of purchase intention. The principal component analysis (PCA) showed factors (overall liking, purchase intention, graphical attractiveness, label clarity) describing and differentiating beer styles. The results provide guidance to beer producers on the factors to be considered when developing an attractive NAB.

## 1. Introduction

The global nonalcoholic beer (NAB) market is projected to expand significantly from USD 22 billion in 2022 to nearly USD 36 billion by 2031 [1]. Nowadays, multinational and craft breweries are interested in having NAB in their assortment. This huge increase in NAB production stems from the better‐for‐you (BFY) consumer trend and the integration of responsible alcohol consumption by global manufacturers into their development strategies [2]. In the beer category, “better” means with low or no alcohol content and low calories but with the same taste as alcoholic beer [3]. Companies started highlighting these characteristics in marketing campaigns, positioning NABs as healthier alternatives to alcoholic beers or even soft drinks [4]. According to Bellut and Arendt [5], Millennials and especially Generation Z, recognized as nondrinkers, are a target group for such healthier beverages. Generation Z, consumers born between 1996 and 2010, are sensitive to various sustainability issues, such as the environment and an inclusive society. They are also more health‐conscious than previous generations and more interested in new experiences and tasting new products. Young consumers are developing habits that will be passed on to future generations [6]. Changing consumer habits toward NABs and reducing overall alcohol consumption are a prerequisite for achieving Sustainable Development Goals, especially Goal 3—Good health and well‐being.

The production method of NAB is a crucial factor determining its sensory quality [7]. NAB producers face the challenge of reconstituting the taste, aroma, and overall experience of alcoholic beers [8]. Balancing alcohol content with satisfying flavor remains challenging, as the absence of ethanol in NAB weakens sweetness and overall beer flavor [8, 9]. Historically, the main NAB styles were pilsner and wheat, followed by radlers [10]. As NAB’s popularity and production grew, many different styles were created. Recently, when craft breweries entered the NAB market, the production of NAB with hoppy flavor profiles, more complex and intense than typical lagers, with moderate bitterness, such as India pale ale (IPA), increased [11]. The flavor attributes are of great importance to the overall acceptance of beer by consumers [12, 13].

## 2. Theoretical Background and Hypothesis Development

To succeed in the market, food and beverage companies should thoroughly understand consumer preferences and perceptions of sensory and nonsensory product traits [14]. Novel and rapid sensory profiling methods are used to better understand the consumer perception of foods [15]. The check‐all‐that‐apply (CATA) method is simple, versatile, easy to understand, and implement by both trained panels or untrained consumers [15, 16]. The CATA can discriminate the sensory attributes even to complex products such as alcoholic beer [17], NAB [18], or wine [16]. In the CATA method, consumers are given a predefined list of potential attributes and terms, and are asked to select those that describe the product [19]. It can be combined with consumer acceptability, known as overall liking [20], defined as a holistic hedonic response in which the consumer evaluates appearance, aroma, taste, or texture. It is a common way to determine consumer acceptability of foods [21].

Understanding how product‐related cues shape expectations and perceptions is key to meet changing consumer needs and enhancing their experience. Therefore, the study aimed to evaluate Polish young consumers’ perception of NABs available on the market and to examine the impact of brand and label on consumer acceptability. Although numerous studies on no‐ and low‐alcohol beverages have been published, the existing literature is dominated by technological research focusing on dealcoholization processes and on general product acceptance. In contrast, relatively few studies address NAB specifically, particular consumer groups, or the influence of external factors such as branding and labeling on consumer decisions. As noted by Waehning and Wells [22], further research is still needed as providing an analysis across different nationalities and geographical regions will allow for better understanding NAB consumers, especially in the context of the global development of the NAB market.

Sensory characteristics are key factors in consumer choice and overall liking determined by the perception of food’s sensory quality [23, 24]. The sensory quality of NABs is still a great challenge for brewers. For years, NABs were criticized for different flavors than conventional ones and a mild overall flavor [8]. Canadian consumers, for example, described NABs as watery and with bland flavor, generally preferring conventional beers [18]. The main reason for rejecting NAB by South Korean beer drinkers was “it does not taste good” [25]. Porretta and Donadini [26] reported that the flavor is in the top three factors affecting consumer choice of NABs. Research of Lafontaine et al. [13] revealed that American consumers preferred commercial NABs with citrusy, tropical, and stone fruit aromas and those perceived as more like soda and sparkling flavored water. When defining sensory quality from a taste perspective, bitterness is a factor differentiating beer styles and influencing consumer preference. American consumers were generally not satisfied with NAB styles perceived as more beer‐like in terms of aroma (i.e., skunk, malty, stale), taste, and mouthfeel (i.e., bitterness), but satisfied with lager‐type and wheat styles recognized as sweeter with fruity and honey aromas [12]. Based on previous findings, we hypothesize that:

 Hypothesis 1. The sensory characteristics of NABs influence Polish young consumers’ preferences and overall liking.

Consumer food preferences and choices depend not only on intrinsic factors like sensory properties but also on extrinsic cues such as brand, price, nutritional information, attitudes and beliefs, convenience, health properties, or previous experience [27]. These extrinsic factors relate to the product but are not physically part of it. In a typical purchasing situation, consumers cannot taste or smell the product before buying it, so they rely on the label information or its functionality. Consumers treat this information as an indicator of what is inside the packaging [28]. Before tasting a product, consumers have an idea of its sensory characteristics and even how much they will like it. Previous product experience can shape consumer expectations [29], which in turn affect their consumption experience. In the absence of information, consumers evaluate foods according to their preferences and sensory attributes, and when the information is disclosed, their choices may change [30–32]. The effect of information on liking after tasting is described as a disconfirmation phenomenon regarding consumer expectations toward products. Both positive and negative disconfirmation may affect consumer perception of sensory attributes, and thus product acceptance [33]. Asioli et al. [34] reported that information has a greater impact on consumer behavior when it is closely associated with sensory attributes under investigation. Therefore, we believe that:

 Hypothesis 2. Extrinsic cues affect the perception of sensory characteristics of NAB by Polish young consumers (blind and informed conditions).

Packaging is the first impression consumers have about foods, which determines the likelihood of purchase. The label is a visual communication allowing consumers to get all the necessary information about the product, especially those that may influence their product evaluation and purchase choice. A product label can influence the perception of the quality and value of a product or can signal the authenticity or tradition. A well‐designed and esthetic label can make the purchase process more pleasant and enjoyable, and the product more appealing [35]. Product brand is one of the pieces of information visible on the label, and brand familiarity may be an important factor affecting not only purchase decisions but even sensory perception [36]. Beneke and Trappler [37] found a strong relationship between brand name and perceived product quality. Gacula et al. [38] reported that consumers were less critical in their evaluation when products were identified by brand names. These could be explained by a strong relationship between brands and emotions [39]. Consumers have stronger emotional affection for packaging than for sensory attributes, and emotions may be better predictors of food choices than liking ratings alone. Certainly, positive emotions can influence consumer liking [40, 41], but reactions to information may vary by product type. Since labels have a significant impact on consumers’ decisions, it is essential to make them truthful. Consumers who have a positive experience with a product may be more inclined to purchase the product again. False and misleading labels can destroy a brand’s reputation and lead to a loss of consumer credibility [42–44]. Thus, our third hypothesis states that:

 Hypothesis 3. Label of NAB influences consumers’ purchase intentions.
   Hypothesis 3a. Consumers prefer well‐known brands of NAB.
   Hypothesis 3b. The graphical attractiveness of the NAB label influences consumers’ purchase intentions.
   Hypothesis 3c. Consumers are more willing to buy NAB with a clear and accurate label.

**FIGURE 1:**
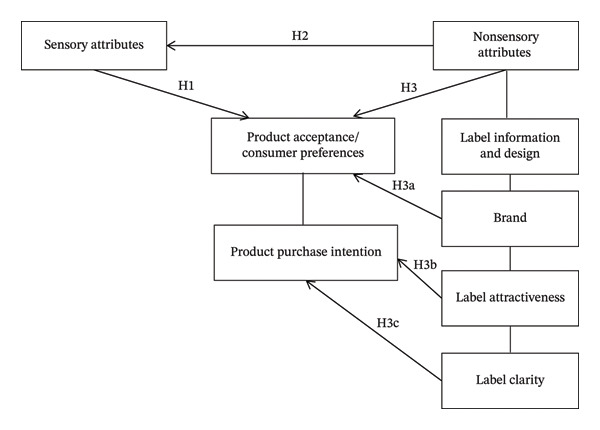
Conceptual framework of the study.

This research was based on a conceptual framework summarized in Figure 1.

## 3. Materials and Methods

### 3.1. Participants

The sensory experiment with consumers took place in a sensory testing laboratory in February–March 2022 at the Poznań University of Economics and Business (PUEB), in accordance with ISO 8589:2007 [45] requirements and with other relevant institutional guidelines and regulations. A total of 61 untrained consumers belonging to Generation Z, aged between 18 and 25, 46% of females and 54% of males, took part in this study (Table 1). The sample size is consistent with recommendations for central location consumer tests and typical comparative sensory studies [46]. This age group is particularly interesting because it is often the time when people start developing their lifetime habits. Participants were recruited using peer‐to‐peer contacts and social media. The experiment ensured anonymity. Each participant gave informed consent by confirming the terms and conditions before taking part in the sensory experiment. After the completion of each session, all participants were given a small gift for their participation in the study. They were instructed to refrain from discussing the details of the study after leaving the testing room.

**TABLE 1:** Sociodemographic characteristics of participants.

Feature	Category	Frequency	Percentage
Gender	Female	28	45.9
	Male	33	54.1

Place of residence	Country	16	26.2
	City below 50,000 citizens	6	9.8
	City between 50,000 and 150,000 citizens	7	11.5
	City between 150,000 and 500,000 citizens	5	8.2
	City over 500,000 citizens	27	44.3

Education	Secondary	42	68.9
	Higher	19	31.3

Nonalcoholic beer consumption	Yes	57	93.4
	No	4	6.6

### 3.2. Products

Different NABs (*n* = 13) from the EU producers were selected (Table 2). Samples were divided into three groups, based on style as (i) IPA: Free Way, Free IPA, Miłosław IPA, and Żywiec Sesyjne IPA; (ii) lager: Tyskie, Żywiec, Lech, Žatecky, and Okocim; and (iii) wheat: Benediktiner, Erdinger, Miłosław, and Paulaner Hefe‐Weissbier, named in the text as: Free Way, Free IPA, Miłosław IPA, Żywiec IPA, Tyskie, Żywiec, Lech, Žatecky, Okocim, Benediktiner, Erdinger, Miłosław, and Paulaner, respectively. The selection was based on product popularity, availability, and affordability, ensuring it reflects the range of options accessible to Polish consumers. Samples were kept at 4°C until testing, but no longer than 3 weeks.

**TABLE 2:** Description of the selected beers.

Product	Style	Alcohol (%)^1^	Picture presented to the participants
Free Way	IPA	0.5	

Free	IPA	< 0.5	

Miłosław IPA	IPA	< 0.5	

Żywiec IPA	Session IPA	0.0	

Benediktiner	Wheat	< 0.5	

Erdinger	Wheat	< 0.5	

Miłosław Pszeniczne	Wheat	< 0.5	

Paulaner Hefe‐Weissbier	Wheat	0.0	

Tyskie	Lager/Pilsner	0.0	

Żywiec	Lager/Pilsner	0.0	

Lech	Lager/Pilsner	0.0	

Žatecky	Lager/Pilsner	0.0	

Okocim	Lager/Pilsner	0.0	

^1^declared on a label.

### 3.3. Design and Procedure

In the first part of the experiment, all participants were asked to answer questions about their sociodemographic characteristics and whether they had ever drunk NAB. They were informed about the product, instructed on the testing procedure, and filling out paper forms. They were given a tray with 3 or 4 different NAB samples (70 mL) served at approximately 10°C in small, identical glasses covered with a glass lid. Samples were poured slowly to reduce the loss of carbonation. They were presented in monadic sequence, coded with random three‐digit numbers, following a balanced randomization order (Williams’ Latin square) to avoid the influence of sample ordering [47]. New bottles were opened for the different sessions. If more than one package of the same beer was served to consumers, multiple bottles were opened and combined into one serving glassware to avoid uneven flavors. Brand names and specific information about the products were unknown to the consumers at the first stage of the experiment to minimize potential bias and allow a more authentic assessment of the intrinsic quality of all products. Participants first assessed the liking of the quality attributes on a 9‐point hedonic scale, where 1 indicated “extreme dislike” and 9 indicated “extreme like,” and then determined the sensory characteristics of NABs using the CATA questionnaire. In a familiarization session, participants received comprehensive instructions about the CATA task and the testing process. The questionnaire consisted of 17 terms, each related to different sensory attributes. These attributes were derived from an extensive literature review and were further refined by a panel of trained and experienced experts from the PUEB. The attributes included in the CATA were randomized to ensure the validity of our questionnaire and mitigate any potential bias that could arise from the presentation order [47]. The evaluation process was structured sequentially, guiding participants to assess the aroma, taste, color, and clarity of samples.

In the second part of the experiment, participants saw NAB brand names and photos of labels and packaging (Table 2). They were then served a tray with known samples and assessed labels’ attractiveness and clarity on a 5‐point Likert scale, ranging from “definitely not” (1) to “definitely yes” (5). Moreover, they determined the overall liking again using a 9‐point hedonic scale. The experiment ended with a “willingness‐to‐buy” question, using a scale ranging from “I would definitely not buy this product” (1) to “I would definitely buy this product” (5).

Our experiment consisted of three separate sessions, each divided into two parts (blind and informed conditions). At each session, 3 or 4 different NABs were evaluated. During all sensory evaluation sessions, consumers were offered either still or sparkling water to cleanse their palates, along with dry, unsalted breadsticks to minimize carry‐over effects from one sample to another. The interval between sessions was one week.

### 3.4. Data Analysis

Statistical analyses were performed using XLSTAT 2023.1.6. and Statistica 13.3 software. The critical significance level was *α* = 0.05. Analyses of variance (ANOVA) were applied to hedonic liking data (overall liking, color, clarity, aroma, and taste). For hedonic liking comparisons, the study employed a within‐subject (repeated‐measures) design, as each consumer evaluated multiple NABs. Such a design is commonly applied in comparative consumer studies and is considered appropriate for sample sizes of 50–80 participants, providing sufficient sensitivity for detecting between‐product differences [46, 48, 49]. The Tukey HSD post hoc test was used to verify the significance of differences between mean values. Student’s *t*‐test was used to detect differences between blind and informed overall liking.

The correspondence analysis (CA) was carried out to analyze the results obtained using the CATA method. Cochran’s *Q* test was applied to CATA counts to determine significant differences in consumer perception of attributes of the beers tested in terms of their styles. If significant differences were found among the variables, post hoc multiple pairwise comparisons were carried out using McNemar’s test with Bonferroni alpha adjustment. The CA was based on chi‐square distance.

The multiple linear regression (MLR) analysis was applied to investigate the impact of label attractiveness and clarity (independent variables) on consumers’ purchase intentions (dependent variable). The model goodness of fit was assessed using *R* ^2^ and adjusted *R* ^2^ values. Multicollinearity between predictors included in the MLR model was assessed using the variance inflation factor (VIF) and tolerance value − low VIF (1.002) and high tolerance value (0.998) indicated that multicollinearity was not a concern.

The principal component analysis (PCA) was used to determine the relationships between parameters (overall liking, purchase intention, graphical attractiveness, and label clarity) and to identify the factors that best describe and differentiate beer styles.

## 4. Results

### 4.1. Effect of Sensory Characteristics of NABs on Consumer Preferences and Overall Liking

The CATA analysis was carried out to verify whether the sensory characteristics affect the young consumers’ perception of NAB (Hypothesis 1). Having 17 different terms, consumers usually used between 2 and 7 terms to describe NABs under study. The percentage of terms ranged from 0% to 26% (Table 3), demonstrating that some terms were product‐specific. Significant differences (*p* < 0.001) in the frequency of the CATA terms were observed between samples, suggesting that consumers distinguished the sensory characteristics of evaluated beers. The exception was the evaluation of sour taste, for which the differences between samples were not significant (*p* > 0.05). This variable was omitted to avoid further problems with a noisy attribute [50]. The terms “refreshing,” “clear,” “fruity,” “bitter,” “malty,” “aromatic,” and “cloudy” were the most frequently chosen (Table 3). This variety of terms underlines the complex sensory expectations and consumer experiences associated with NAB.

**TABLE 3:** Frequency (%) of the terms used by consumers to describe NAB samples (CATA analysis).

Attributes	Free Way	Free IPA	Miłosław IPA	Żywiec IPA	Tyskie	Żywiec	Lech	Žatecky	Okocim	Benedictiner	Erdinger	Miłosław	Paulaner
Refreshing	1.1	1.0	9.2	9.2	7.4	18.4	16.8	9.7	18.9	9.8	9.2	7.2	5.7
Fruity	18.0	10.4	10.4	17.9	1.2	1.6	6.7	0.8	5.9	7.8	8.5	7.9	8.0
Sweet	10.2	5.2	10.4	12.8	2.1	2.5	8.7	2.1	3.6	9.8	7.7	0.7	4.2
Bitter	10.2	8.4	6.1	8.3	10.7	12.3	3.8	5.0	3.6	3.9	6.3	13.4	5.4
Citrus	12.4	12.0	13.2	2.8	2.1	1.6	3.8	0.0	1.8	3.5	9.2	11.2	3.8
Candy	3.0	1.6	3.8	2.8	0.0	0.0	1.4	0.0	0.5	2.0	0.0	1.4	0.8
Malty	3.4	5.2	2.8	4.1	19.0	11.5	7.7	16.0	12.2	9.8	7.4	5.8	8.8
Astringency	2.3	6.5	1.0	2.3	6.6	4.1	3.4	4.6	2.7	3.1	4.4	7.2	4.6
Bitterness hops	5.3	10.1	7.1	2.8	9.5	12.3	6.3	5.0	11.7	3.5	3.0	4.7	4.6
Metallic	1.5	3.6	0.3	3.2	4.1	1.6	1.0	7.1	2.7	3.1	2.2	5.1	3.1
Clear	1.1	0.0	0.8	3.2	24.0	20.9	26.0	21.8	23.0	7.5	4.1	4.0	1.1
Cloudy	18.8	16.9	14.8	14.2	0.0	0.4	0.0	0.0	0.0	1.6	12.5	6.1	15.7
Aromatic	5.3	9.4	14.0	3.7	6.2	6.6	8.7	15.5	9.5	12.9	4.8	4.0	2.3
Faint	5.3	5.8	2.8	6.4	2.5	0.4	1.4	5.0	0.5	0.8	3.0	4.0	5.4
Bread	0.0	0.0	0.0	0.0	0.0	0.0	0.0	0.0	0.0	11.4	11.4	2.9	14.6
Spiced	0.0	0.6	1.0	0.0	0.0	0.0	0.0	0.0	0.0	6.3	3.7	8.3	6.1
Sour	2.3	3.2	2.3	6.4	4.5	5.7	4.3	7.1	3.6	3.1	2.6	6.1	5.7

The CA was carried out for the visualization of the relationship between consumer perception of sensory attributes and beer styles. It was found that 72.9% of the total data variance was explained by the first two factors (Figure 2(a)). According to consumer evaluations, the samples were grouped into three main categories reflecting different beer styles. Consumers often described lager‐style beers as “refreshing,” “clear,” “malty,” “bitterness hops,” and “aromatic,” in line with their expected profiles. IPA‐style beers were mainly characterized as “fruity,” “citrus,” “sweet,” and “cloudy,” reflecting consumers’ appreciation of the complex flavor characteristic of this style. Wheat NABs were mostly associated with the descriptors “bread” and “spiced.” Figure 2(b) shows the relationship between the overall liking and the 16 sensory attributes used to describe NABs. It was found that the perception of sensory attributes of NABs influenced consumer preferences. The overall liking was significantly positively correlated with “refreshing,” “clear,” and “aromatic” attributes, and negatively with “cloudy” and “faint.” The “positive group” of attributes was associated mainly with lager‐style beers, while the “negative group”—with IPA‐style beers. No significant correlation was found for wheat beers. These results confirm Hypothesis 1 and suggest that Polish young consumers perceive nonalcoholic lager beers better than IPA or wheat beers.

FIGURE 2Multiple factor analysis (MFA) of CATA data: (a) NABs and sensory attributes, (b) the overall liking and sensory attributes used to describe NABs.

**Figure:**
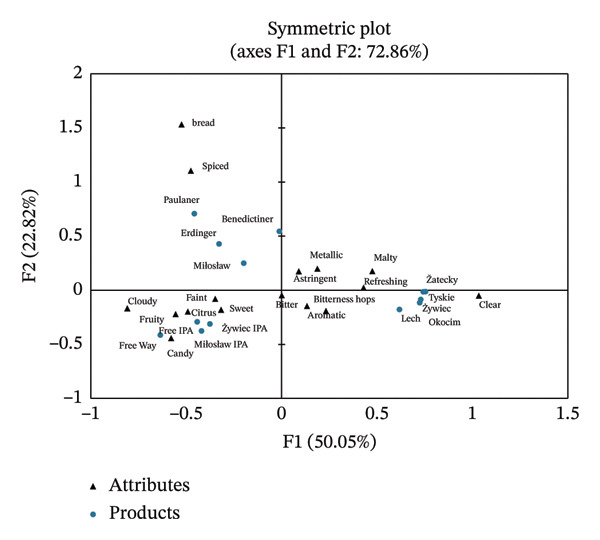
(a)

**Figure:**
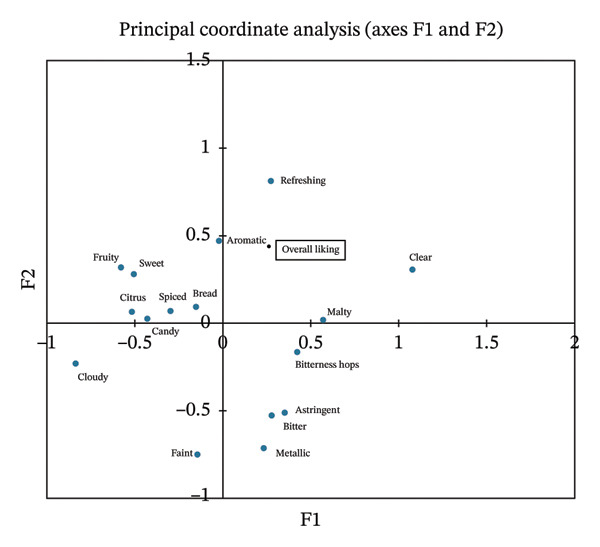
(b)

The impact of extrinsic cues on the young consumer’s perception of the sensory characteristics of NABs was investigated in blind and informed conditions, in which brand information was disclosed. This approach allowed us to understand not only the intrinsic sensory appeal of NABs but also the impact of external cues (brand and label familiarity) on consumer preferences.

Consumers evaluated the color, clarity, aroma, and taste (Figures 3(a), 3(b), 3(c)) of beers without brand or packaging information, relying only on their sensory impressions. A significant effect of beer style and brand on the degree of acceptance of the organoleptic characteristics of NABs was found (*p* < 0.05). Among IPA beers (Figure 3(a)), the color and clarity of Żywiec IPA were the most acceptable to consumers, scoring 5.4 and 5.1, respectively. In terms of taste, consumers rated this beer (score 5.5) and Miłosław IPA (score 5.8) the best. In terms of aroma, Free Way beer was rated the highest (score 6.2). For lager beers (Figure 3(b)), consumers rated Żywiec, Žatecky, and Okocim the highest in terms of desired color and clarity. The acceptance level of the taste of these beers was similar, except for Tyskie, which was rated the lowest (score 3.1). The aroma of this beer was also rated the lowest (score 4.2), but the result was not significantly different from Żywiec (score 4.9) and Žatecky (score 5.0) (*p* > 0.05). Among wheat beers (Figure 3(c)), consumers rated Benediktiner the highest in terms of all organoleptic attributes. The relationship between overall liking and liking of individual organoleptic attributes of NABs under study was also investigated. A positive moderate correlation was found between the overall liking and liking of color, clarity, and taste (*r* = 0.695; *r* = 0.764; *r* = 0.754, respectively). No significant correlation was found between overall liking and aroma, suggesting that aroma is not an important attribute for young consumers of NABs.

FIGURE 3Mean values of sensory attributes of NABs in blind condition: (a) IPA beers, (b) lager beers, (c) wheat beers. Values are means ± standard error of the mean (SEM); (A–C) mean values with different letters were significantly different according to the Tukey test (*p* < 0.05).

**Figure:**
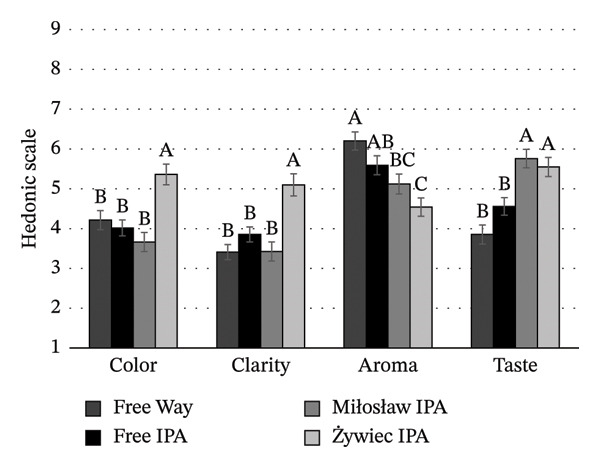
(a)

**Figure:**
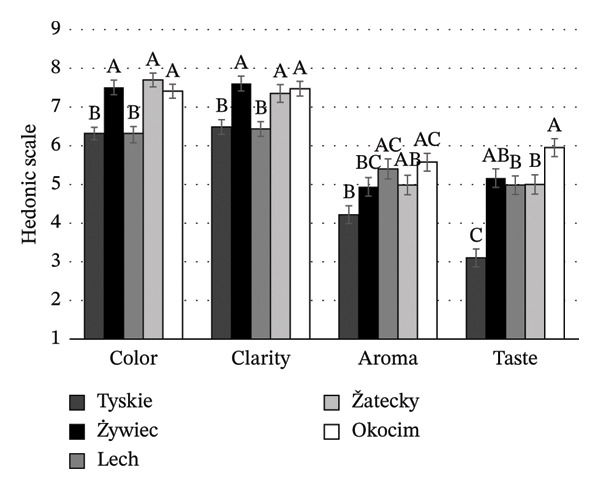
(b)

**Figure:**
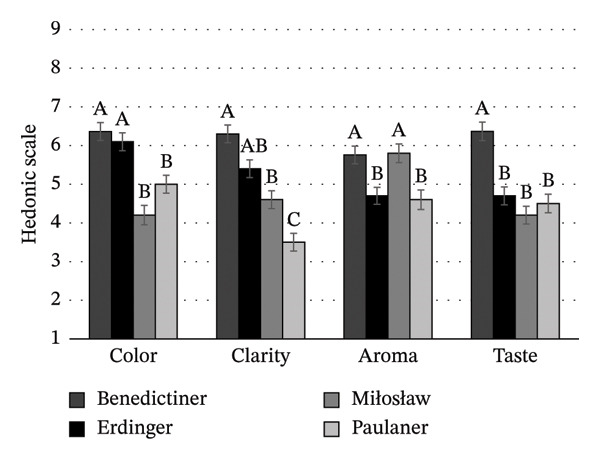
(c)

**FIGURE 4:**
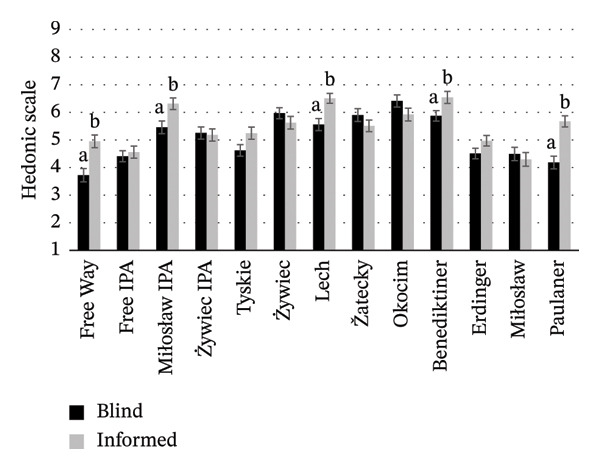
Mean scores for the overall liking of NABs evaluated in blind and informed conditions. Values are means ± standard error of the mean (SEM); (a, b) mean values with different letters were significantly different according to the Student t‐test (*p* < 0.05).

To assess how brand familiarity and visual presentation affect liking, consumers re‐evaluated the beer with brand and label information. Figure 4 shows NABs overall liking in blind and informed sessions. A positive correlation (*p* < 0.05) was found between overall liking in blind and informed conditions (*r* = 0.710). The transition from blind to informed conditions revealed interesting changes in consumer preferences. For example, some beers that were perceived more favorably in the blind conditions than other beers (Miłosław IPA, Lech, Benediktiner) continued to be rated highly after providing brand information. This suggests that a well‐perceived brand can confirm or even improve the perception of the sensory characteristics. On the other hand, some beers that did not score as high on sensory appeal (Free Way and Paulaner) received better ratings in the informed conditions, indicating that attractive branding and packaging can positively influence consumer perception and potentially compensate for the product’s sensory deficiencies. These differences underline the power of a brand and its label in shaping consumer expectations and experiences. All the above support Hypothesis 2 and justify Hypothesis 3.

### 4.2. The Effect of Brand Familiarity, Consumption Experience, and Label of NABs on Purchase Intention

In order to verify Hypothesis 3, beer labels were presented to consumers who were asked about: (1) brand familiarity, (2) whether they had ever consumed these beers, and (3) how they perceived the labels in terms of their graphical attractiveness and clarity. They also declared their purchase intentions.

**FIGURE 5:**
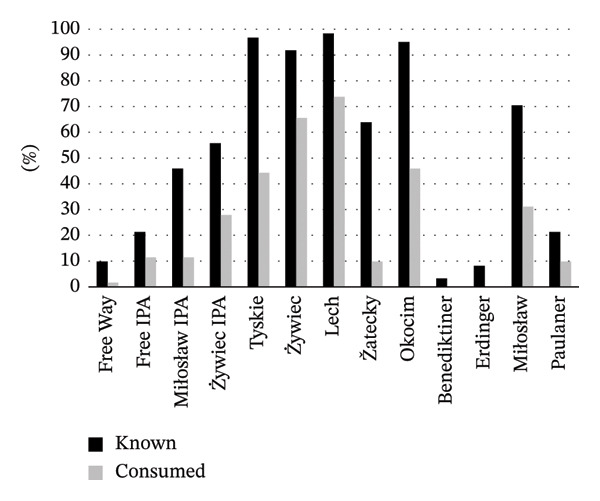
Consumer declarations on brand familiarity and their experience of consuming NAB (%).

Consumer declarations on brand familiarity and their consumption experience showed that lager beers were the most recognizable and consumed style (Figure 5). Consumers were widely familiar with the Tyskie, Żywiec, Lech, and Okocim brands: approximately 97%, 92%, 98%, and 95% of respondents, respectively, declared familiarity. These beers were also consumed by most of the respondents (44% for Tyskie, 66% for Żywiec, 74% for Lech, and 46% for Okocim). The exception was Žatecky, which was recognized by 64% and consumed only by 10% of respondents. This means that a large number of those who recognized the brand also decided to purchase it. These high indications may suggest an appropriate and effective marketing strategy adopted by the manufacturer of a particular brand. In contrast, IPA brands (Miłosław IPA and Żywiec IPA), which were known to 46% and 56% of consumers, respectively, were not willingly purchased (12% and 28% of respondents, respectively, declared their consumption). This suggests that IPA beers may generate interest, but this interest does not translate into purchase decisions. Wheat beers were generally less familiar to consumers, except for the Polish brand Miłosław (71% of consumers declared familiarity and 31% declared consumption). German brands, Benediktiner, Erdinger, and Paulaner, were known by only about 3%, 8%, and 21% of the surveyed consumers. Their consumption rates were also low or almost negligible, indicating their niche status in the Polish market.

A strong correlation was found between the variables “known” and “consumed” (*r* = 0.896), indicating that brand familiarity significantly affects consumption behavior; when consumers recognize or are aware of beer, they are more likely to try it. The above results confirm Hypothesis 3a.

**FIGURE 6:**
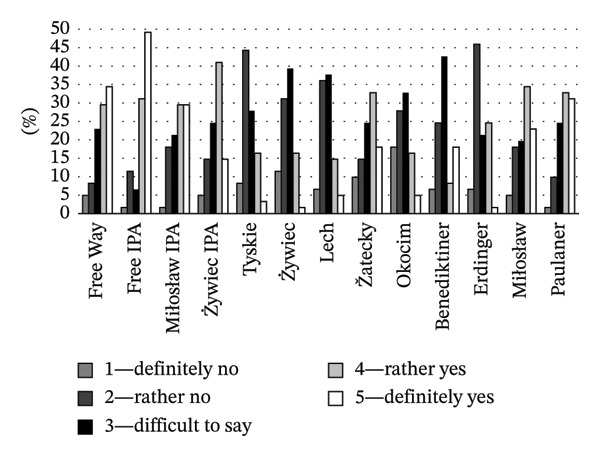
Consumer opinion about the graphical attractiveness of the NAB label (%).

Consumer opinions about the attractiveness of NAB labels are shown in Figure 6. Free IPA stood out in this category, as 80% of consumers rated its label as “rather and definitely attractive,” indicating strong appreciation of the label’s vivid and modern design, which can contribute to beer purchase. On the other hand, Żywiec IPA, although more popular among respondents (28% had ever drunk this beer; Figure 5), its label was considered attractive by 56% of respondents. This suggests that although the label of this beer has some appeal, it lacks a striking visual impact like that of Free IPA. In contrast, lager beers, despite their established presence in the market, showed poor scores in terms of packaging attractiveness, with a maximum of 21% of respondents finding their labels attractive, except Žatecky (51%). Wheat beers, valued by many consumers for their unique taste, were rated more positively (57% for Miłosław and 64% for Paulaner), highlighting their visual appeal, probably due to the traditional design attracting consumer attention.

**FIGURE 7:**
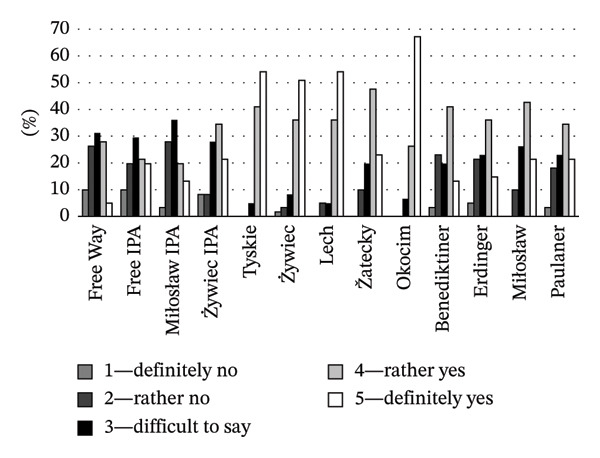
Consumer opinion about the clarity of the NAB label (%).

The results of the present study showed that the clarity of NAB labels (defined as easy to read, clear, and not overloaded with optional information) was generally rated well, with higher ratings observed for acclaimed lager beer brands, ranging from 71% for Žatecki to 95% for Tyskie (Figure 7). This demonstrates broad consumer acceptance, highlighting effective communication through their labels. The labels of lager beers were not rated high in terms of attractiveness (positive ratings from 18% to 51%; Figure 6), indicating a potential area for improvement in visual appeal. Over 50% of respondents indicated that the labels of wheat NABs clearly present product details. Both Miłosław and Paulaner were not only transparent in terms of information on the label (64% and 56% positive answers, respectively; Figure 7) but also scored high in terms of visual appeal (57% and 64% of respondents, respectively; Figure 6). It suggests that the labels of these beers successfully integrated clarity and attractiveness. For IPA beers, the clarity of labels was rated positively (“rather and definitely clear”) by 33%–56% of respondents. Positive ratings for the attractiveness of IPA beer labels ranged from 56% to 80% (Figure 6). This spread of ratings highlights a significant difference in the effectiveness of label communication between the beers of this style.

**FIGURE 8:**
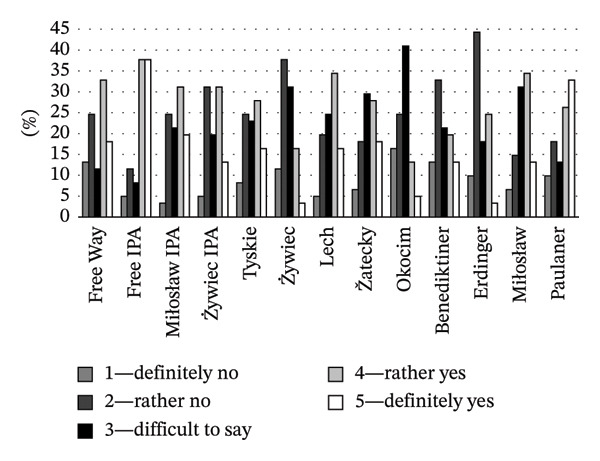
Consumer purchase intention of NABs (%).

When considering the purchase intentions across different beer types, varying relationships were observed, reflecting various factors influencing consumer choices. Statistical analysis of the results showed the influence of beer style on purchase intentions (*F* = 7183, *p* = 0.001). The relationship between the attractiveness of a beer label and the purchase intention can be seen in the example of IPA beers. The high proportion of consumers who found the IPA beer label attractive (56%–80% of respondents; Figure 6) is reflected in high purchase intention scores (44%–75%; Figure 8), suggesting that attractive packaging can effectively result in increased beer sales. The results for lager beers were quite diverse. For lager beers (Tyskie, Żywiec, Lech, and Okocim), purchase intentions ranged from 18% to 51%, reflecting moderate interest despite generally high brand familiarity (Figure 5). Moreover, only 18%–21% of consumers found the packaging of these brands attractive (Figure 6). On the other hand, the clarity of the labels of these beers was rated positively by 87%–95% of respondents (Figure 7). It suggests that clear and simple labels contribute significantly to purchase decisions, helping to mitigate the lower visual appeal. Although wheat beers were generally less popular among Polish consumers than IPA and lager beers (Figure 5), the consumer purchase intentions were comparable with other beers (Figure 8). Improving the attractiveness and clarity of wheat beer labels can effectively convert interest into sales.

The MLR analysis was conducted to investigate the impact of label attractiveness and clarity on consumers’ purchase intentions. The regression model explained 36.84% of the data variance, as indicated by the *R* ^2^ of 0.3684, with the adjusted *R* ^2^ of 0.3668. The model was statistically significant (*F*(2,790) = 230.40, *p* < 0.0001), indicating reliable predictive power. Graphical attractiveness of the label was a strong predictor of purchase intention (*b* = 0.587, *p* < 0.0001). Label clarity was also positively related to purchase intention, though to a lesser extent (*b* = 0.194, *p* < 0.0001). The regression equation is as follows:

 purchase intention = 0.548 + 0.587 × graphical attractiveness + 0.194 × label clarity.

These results confirm Hypotheses 3b and 3c.

**FIGURE 9:**
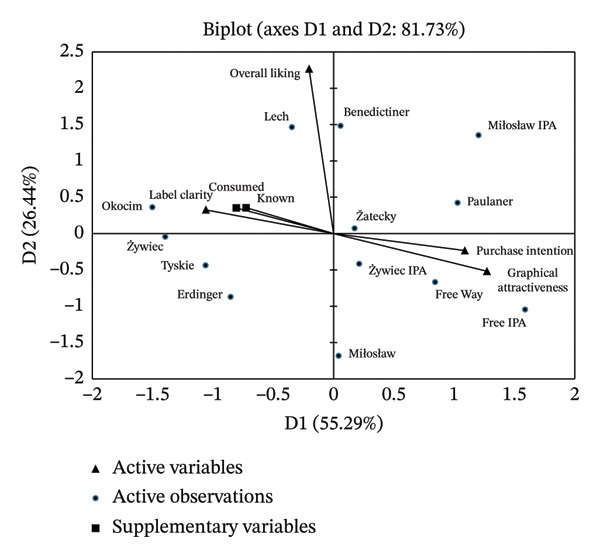
PCA biplot of NABs and factors (overall liking, purchase intention, graphical attractiveness, and label clarity). Supplementary variables: “known” (brand familiarity) and “consumed” (beers actually consumed by respondents).

PCA was conducted to complement the above findings and provide a more comprehensive illustration of which factors (overall liking, purchase intention, graphical attractiveness, and clarity of the label) describe and differentiate the beer styles. The first two principal components explained 81.7% of the total data variance (Figure 9). The CATA analyses (Figure 2) showed that overall liking was influenced by sensory attributes, with “refreshing,” “clear,” and “aromatic” attributes correlating positively. Lager‐style beers were primarily associated with “positive” sensory attributes supporting Hypothesis 1. The PCA confirmed that most lager beers were linked to overall liking, as well as to label clarity. Wheat beers, like IPA beers, were generally perceived positively by consumers due to their attractive labels, which significantly influenced consumers’ purchase intentions. However, exceptions such as Benediktiner and Erdinger point to differences in consumer preferences. Benediktiner was well‐scored (overall liking 5.9 and 6.5 in blind and informed conditions, respectively; Figure 4) despite its lower visual appeal and purchase interest. This indicates that sensory attributes or prior experience may outweigh label effects. Sensory attributes such as “refreshing,” “clear,” and “aromatic” were strongly related to overall liking, while purchase intentions were more influenced by label attractiveness, particularly for IPA beers.

The complementary variables “known” and “consumed” showed strong positive correlations with label clarity (0.827 and 0.846, respectively). The integrated analyses of MLR and PCA confirm the importance of both sensory and extrinsic attributes in shaping consumer preferences and purchase intentions, supporting Hypotheses 1, 3b, and 3c.

## 5. Discussion

### 5.1. Theoretical Contribution and Implications

Sensory perception is the first stage of consumer interaction with a product. The product sensory attractiveness plays a crucial role in the perception, purchase decision, consumption, and satisfaction of consumers. This complex phenomenon engages all the senses: sight, smell, taste, touch, and hearing [51, 52], but numerous studies have shown that sight and smell have a particularly strong influence on consumers’ first impressions. The packaging and visual attributes, such as the appearance, color, and shape of the product, can increase perceived value and quality, influencing purchase intentions and product acceptance. Aligning with personal or cultural preferences, initial visual appeal can shape consumer expectations and influence the expected taste and overall liking of foods [52–54]. In the context of beer, color is a particularly important visual attribute, as it can influence the perceived flavor intensity and quality. Consumers tend to associate certain colors with specific taste profiles; e.g., darker beers are usually perceived as richer and more flavorful [55–57]. Clarity is also important for beer consumers. Some studies showed that consumers generally prefer clear beers, associating clarity with freshness and quality. Cloudy or hazy beers may be perceived as less attractive, although this depends on the beer style and consumer familiarity with the beer. For example, while clarity is preferred in lager beers, the growing popularity of cloudy IPA beers shows that consumers are now more open to cloudy beers, especially craft beers [58, 59].

Our research shows that both sensory attributes and external factors interact to create an overall consumer perception of NAB. The results of the CA showed that Polish young consumers preferred clear, refreshing, and aromatic NABs. These attributes were indicated mainly for lager‐style NABs. Polish consumers less appreciated cloudy and faint beers, mainly associated with nonalcoholic IPA‐style beers. Similarly, Moss et al. [18] reported that Canadian consumers described NABs as watery and bland, highlighting the need for stronger and more flavorful sensory profiles to improve acceptability. Lafontaine et al. [12, 13] showed that American consumers prefer sweet NABs (e.g., radlers and IPA) with citrusy, tropical, and stone fruit aromas. Donadini et al. [60] assessed the perception of specialty beers in Italy, Spain, and Poland. They found that Polish males disliked fruity‐tasting beers, whereas Spanish and Italian males favored them. These differences between consumers of different nationalities confirm the need for further research on consumer preferences for NABs.

The extrinsic cues, such as packaging, labeling, brand, country of origin, price, place of choice/consumption, container type, and other factors, may affect consumer preferences for beer [55, 61]. These external factors influence not only the initial purchase decision but also the overall satisfaction and perception of quality during consumption. Attractive packaging can enhance the perceived value and quality, making the product more appealing to consumers [62, 63]. We found that the knowledge about brand and packaging may improve the perception of sensory characteristics of NAB as well as its overall liking. Moreover, a well‐perceived and familiar brand confirms or even improves the perception of the sensory characteristics of NAB. Consumers who recognize a particular beer are more likely to purchase and try it. Our findings underline the power of branding and packaging in shaping consumer expectations and experiences. This aligns with Thong et al. [64], who found that brand names significantly influence preferences, with international brands often favored. They also noted that well‐established brands tend to enhance the perceived quality and overall attractiveness of the product. Guinard et al. [65] also revealed significant changes in ratings of domestic, imported, or specialty lager beers when consumers were aware of the brand and price. This confirms the importance of nonsensory variables in shaping consumer preferences and evaluations.

The lower familiarity and consumption of some styles or brands of NABs (e.g., some IPA and wheat NABs among Polish consumers) may reflect limited availability or targeted marketing. Accessibility has been identified as an important external factor in some studies, but their results are not always consistent. They show, e.g., that increased availability of a product does not necessarily mean increased sales and consumption [66, 67]. Further studies are required to explore the complex interaction between availability, consumer awareness, and marketing strategies. It would be beneficial to examine how these factors affect consumer perceptions and behavior across demographic groups and regions.

The results of many studies showed the strong link between packaging design and consumer buying patterns [61, 68, 69]. Impulsive purchasing behavior largely depends on the attractiveness of packaging and its presentation. For example, for Italian NAB consumers, packaging was the most important attribute, followed by price, flavor, claims, and color [26]. Barnett and Spence [70] demonstrated that changes in the label’s color and descriptive notes can significantly influence how consumers perceive the taste and quality of the beer. It further emphasized the impact of graphical attractiveness and clarity of labels on consumer behavior.

The design of the label is another factor directly influencing consumers’ purchasing decisions. A well‐designed label is essential for conveying information unique to a particular beer, while simultaneously, it should meet consumer expectations for readability and clarity [71]. The results of the present study confirm that the design of a beer label is important in attracting consumer attention and building a relationship with the product. Polish consumers perceived wheat NABs similarly to IPA beers, associating them positively with their attractive labels. However, exceptions like Benediktiner, which had high overall liking despite low visual appeal, indicate that sensory attributes and personal experiences also play a significant role. Consumers appreciated the clarity of the Erdinger beer label, aligning it more with lager beer. Lager beers were characterized by consumers as having clear labels, but they did not necessarily find them graphically attractive. It reflects consumer preference for clear and informative labels over visually appealing ones. Moreover, the graphical attractiveness of the label was a strong predictor of purchase intention, suggesting that improving label esthetics significantly increases the probability of purchase. These observations are in line with previous studies, showing that packaging influences both sensory and price evaluations [72].

### 5.2. Practical Implications

The findings of our research provide actionable guidance for NAB producers and marketing practitioners. NABs have long been perceived as dull and poorly aligned with the sensory profile of alcoholic beers, which discourages purchase. As demand grows, producers should expand their portfolios and develop NAB styles with clearly optimized sensory profiles. Our results show that NABs perceived as refreshing, aromatic, and clear are more likely to be chosen by Polish young adults. Brewers should therefore prioritize enhancing the freshness cues, aroma intensity, and visual clarity, supported by consumer‐driven sensory profiling. For nonalcoholic IPAs in particular, brewers should reduce excessive sweetness and increase hop and citrus notes to create more balanced, vivid, and flavorful products, addressing perceptions of these beers as boring and lacking freshness. These actions are crucial, as flavor differences influence both consumer liking [72] and choice [62].

Since some consumers appreciate a bitter profile, it is important to ensure a balanced bitterness that does not overshadow other attributes. It can enhance the overall flavor of NAB without reducing its appeal [12]. Based on consumer evaluations, the results of this study can support decisions aimed at improving the sensory quality and market attractiveness of NABs. However, as suggested by Silva et al. [41], it is important to underline that consumer opinion may be based on beer labeling, mainly alcohol‐related information, rather than its actual taste. Therefore, brewers should pay attention to changing consumer conviction and the reputation of NABs, especially since considerable effort is being made to improve taste and aroma.

When consumers are unfamiliar with a product, extrinsic cues, especially the label, play a key role in purchase decisions. Product labels influence not only choice, but also perceived liking and sensory expectations. Brewers and marketing practitioners should therefore clearly communicate the specific characteristics and validated benefits of NABs, with particular attention to health‐conscious consumers. Our results indicate strong demand for more dynamic and innovative packaging, even for well‐known brands. This suggests that nonalcoholic lager producers should redesign visual identities to increase shelf impact, maintain consumer interest, and compete more effectively with visually distinctive IPA products. The combination of label esthetics and clear information can be a driver to gain consumer interest and increase the probability of purchase and consumption rates. In the case of commercial lager NABs, label clarity remains a strategic priority for maintaining consumer loyalty and market presence. Generally, in new product development and brand renewal, a strong effort should be made to design a label that is attractive, informative, clear, and adequate to the product to satisfy consumer expectations.

### 5.3. Limitations and Future Directions

While our findings may have important implications for both theory and practice, it is crucial to acknowledge certain limitations that can guide future research. Our studies were conducted at the university’s sensory laboratory in winter. NABs are rather “summertime” beverages, and therefore, the season and laboratory environment could be the limitations. Young consumers could feel uncomfortable drinking NABs at the university. In laboratory conditions, the pleasure derived from the consumption of NABs may be reduced, and the appreciation of individual sensory attributes may change. The same product is perceived differently depending on the consumption place; it could be perceived as the best in a restaurant and inferior in a laboratory. Because of the context of our experiment, the sensory perception of NABs may vary.

Finally, in our study, 13 different NABs available on the Polish market were chosen. They were both craft NABs, Polish and German wheat NABs, and typical nonalcoholic lagers. Familiarity with the product may have influenced the purchase intentions and thus affected the verification of Hypothesis 3. Although we know the impact of price on consumer choices, we decided not to include this factor in our study. However, when selecting beers, we considered their affordability. The NABs tested varied in price (from about 1 EUR to 3 EUR), and young consumers with limited income may intentionally choose cheaper products. Future research should also include price as a possible factor, or the study should evaluate samples at a similar price.

## 6. Conclusions

Polish young consumers preferred refreshing, aromatic, and clear NABs. In this context, they perceived lager beers better than IPA and wheat beers. Some NABs can be consistently positively perceived by consumers in many aspects, such as attractiveness, liking, and purchase intentions. Other beers may be known and have clear labels, but these aspects do not necessarily result in a positive perception of label esthetic or purchase intention. The integrated analysis of MLR and PCA confirmed the importance of both sensory characteristics and extrinsic attributes (attractiveness and clarity of the label) in shaping consumer preferences and purchase intentions. MLR analysis showed that attractiveness and clarity of label can be good predictors of the purchase intentions of Polish young consumers and can increase consumer interest in NABs. This highlights the need for strategic marketing that combines clear and informative labeling with a graphical appeal to increase consumer interest and producer sales of NABs.

## Author Contributions

Dorota Klensporf‐Pawlik: conceptualization, methodology, resources, writing–original draft, and writing–review and editing. Inga Klimczak: conceptualization, data curation, formal analysis, investigation, methodology, resources, project administration, supervision, validation, visualization, writing–original draft, and writing–review and editing. Iga Rybicka: conceptualization, resources, writing–review and editing. Anna Gliszczyńska‐Świgło: conceptualization, data curation, formal analysis, investigation, methodology, resources, project administration, supervision, validation, writing–original draft, and writing–review and editing.

## Funding

This research did not receive any specific grant from funding agencies in the public, commercial, or not‐for‐profit sectors. It was financed by Poznań University of Economics and Business as part of a subsidy received from the Ministry of Science and Higher Education.

## Ethics Statement

The study and its purpose were thoroughly explained to all participants. Each participant gave informed consent by confirming the terms and conditions before taking part in the study. They were informed of their right to withdraw from the study at any time without providing a reason. The participation was entirely voluntary and anonymous, and the data were re‐identified and reported solely in aggregate form. The research was conducted in accordance with the relevant guidelines and regulations (Committee on Research Ethics at the Poznań University of Economics and Business. The ethical recommendations for researchers, 2025 (https://ue.poznan.pl/uniwersytet/badania-naukowe-na-uep/komisja-ds-etyki-badan-naukowych/)).

## Conflicts of Interest

The authors declare no conflicts of interest.

## Data Availability

The data that support the findings of this study are available from the corresponding author upon reasonable request.
